# Supplementary figures and images for: Identification of a gene expression signature of vascular invasion and recurrence in stage I lung adenocarcinoma via bulk and spatial transcriptomics
Source: bioRxiv. 2024 Jun 10:2024.06.07.597993. Preprint. [Version 1] doi: 10.1101/2024.06.07.597993 (PMC11195124; doi:10.1101/2024.06.07.597993)

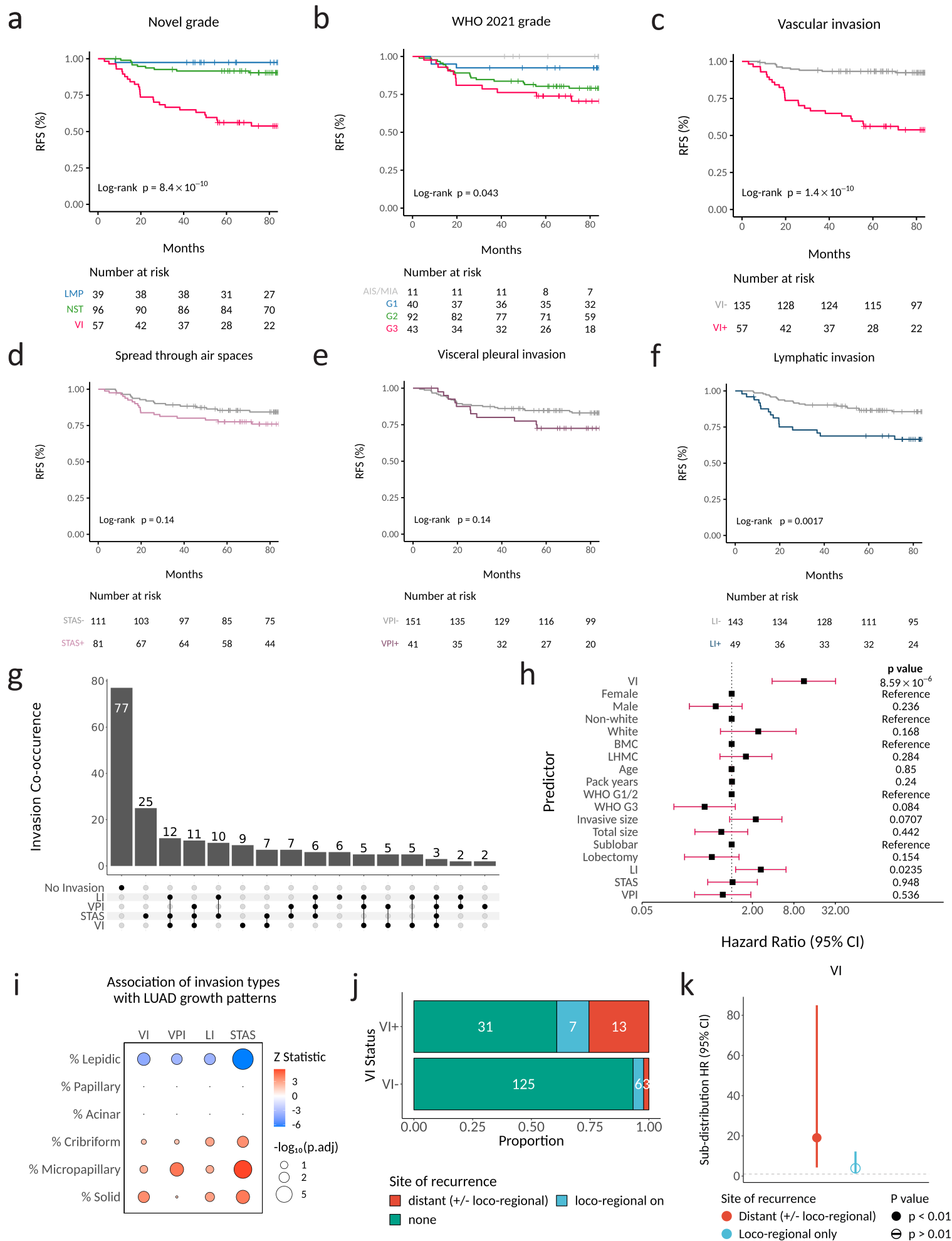

**Extended Data Fig. 1. VI is the stage I LUAD invasion type most associated with recurrence.**

Supplement: Supplement 1 — Extended Data Fig. 1. VI is the stage I LUAD invasion type most associated with recurrence. a. Association of novel grading (n=192 tumors) and b. WHO grading (n=186 tumors) systems with 7-year RFS in the stage I LUAD discovery cohort. Mucinous tumors were excluded from WHO grading. P values were calculated by log-rank test. c-f. Association of VI, STAS, VPI, and LI pathology, respectively, with RFS. g. Co-occurrence of invasion types in the stage I LUAD clinical cohort. h. Association of VI with RFS (n=183 tumors) when controlling for common clinical variables, collection site (LHMC – Lahey Hospital & Medical Center, BMC – Boston Medical Center) and the other invasion types. Patients with missing values for any of the covariates were excluded. P values derived from multivariate Cox regression. i. Association between invasion types and LUAD growth patterns. Bonferroni adjusted p values (p.adj) were calculated by Wilcoxon test j. Proportion of VI- and VI+ cases that recurred at different locations. k. Recurrence site-specific sub-distribution HR for VI (n=185 tumors). Patients with unknown recurrence site were excluded from the analysis. HRs were adjusted for gender, age, pack years, surgical procedure, and collection site. Vertical lines designate the 95% CI. P values were calculated by multivariable Fine-Gray regression. [file media-1.pdf]

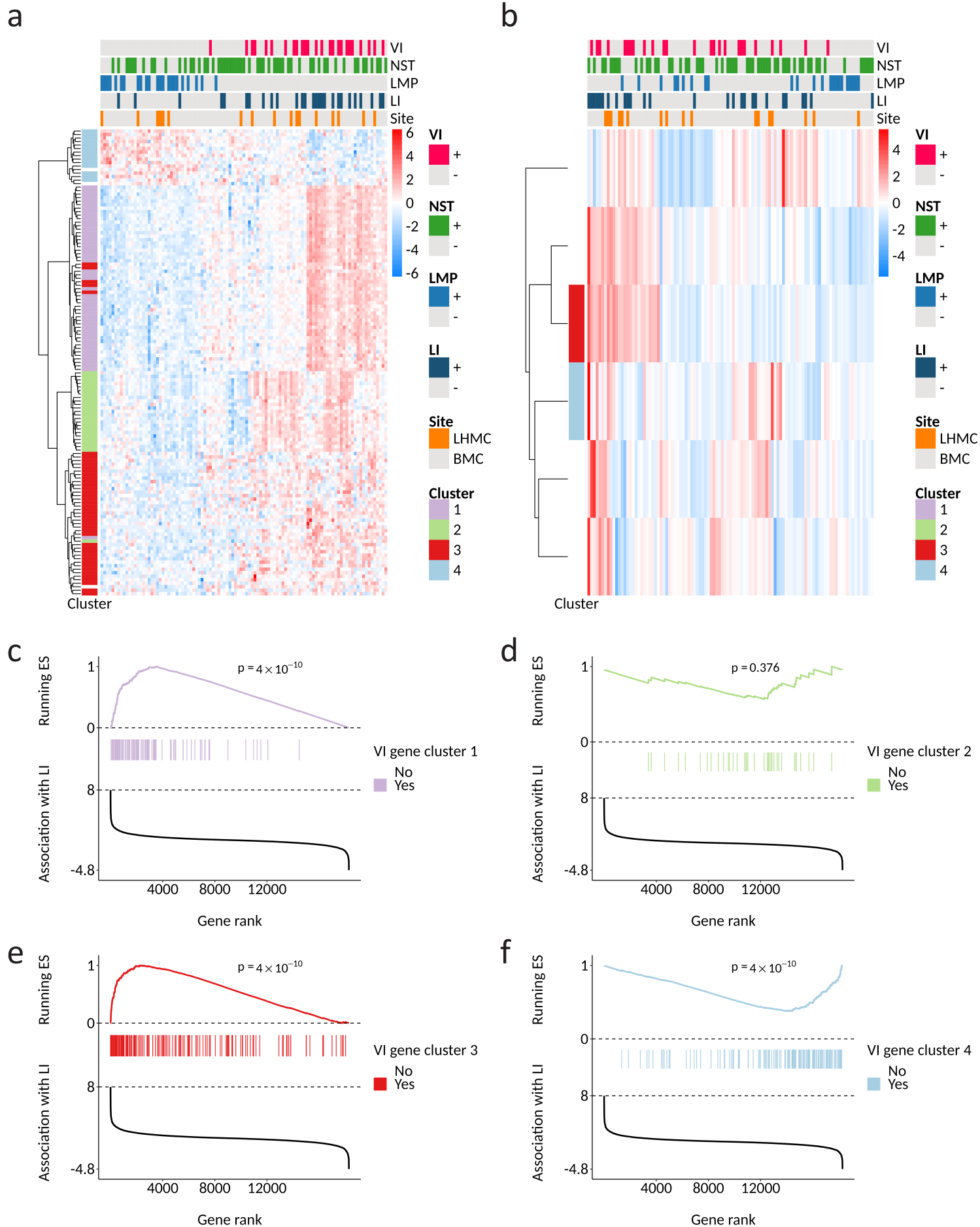

**Extended Data Fig. 3. VI but not LI is associated with expression of tissue remodeling genes.**

Supplement: Supplement 3 — Extended Data Fig. 3. VI but not LI is associated with expression of tissue remodeling genes. a. Co-expression heatmap of 133 genes differentially expressed between VI and LMP (FDR < 0.01) in a discovery cohort of stage I LUAD tumors (n=103), with LI as a covariate. Genes are annotated by the clusters defined in Fig 1b. Heatmap units are log counts per million (CPM) scaled by transcript. VI, vascular invasion; NST, no special type; LMP, low malignant potential; LI, lymphatic invasion. b. Heatmap of 6 genes associated with LI contrasts (FDR < 0.01). Genes are annotated if they belong to one of the clusters defined in Fig 1B. c-f. Gene-set enrichment analysis (GSEA) results of the 4 VI gene clusters against a ranked list of genes ordered by association with LI. P values were calculated by GSEA. [file media-3.pdf]

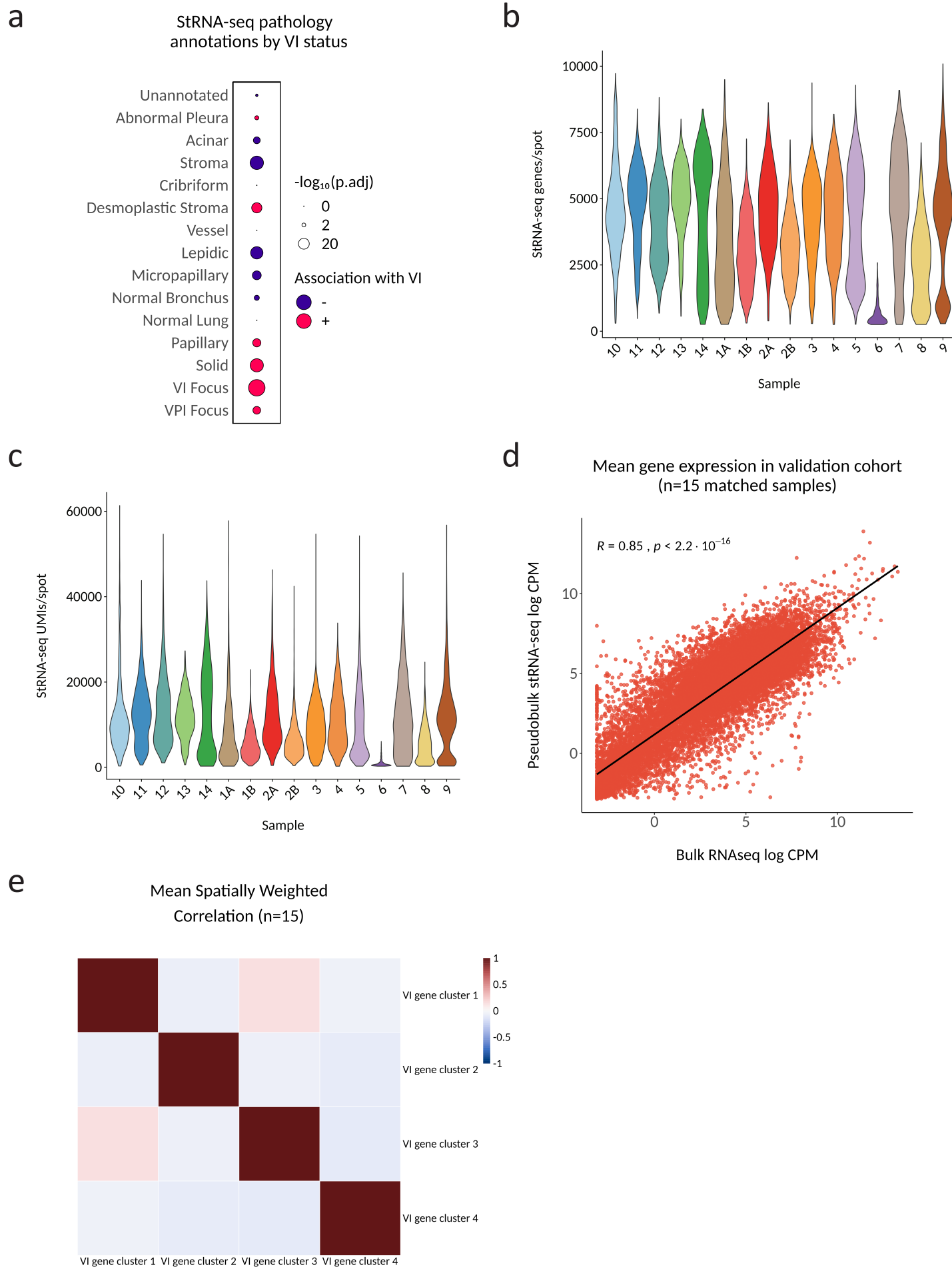

**Extended Data Fig. 4. StRNA-seq quality control and concordance with bulk RNA-seq.**

Supplement: Supplement 4 — Extended Data Fig. 4. StRNA-seq quality control and concordance with bulk RNA-seq. a. The association of spot-wise pathology annotations with tumor-level VI status in stRNA-seq samples that passed QC (n=15) (red = over-represented; blue = under-represented). P.adj values were calculated by chi-square test. b. Features per spot and c. UMI counts per spot by sample in the stRNA-seq data prior to QC (n=16). Sample 6 failed QC and was excluded from downstream analysis. d. Correlation of mean gene expression between pseudo bulked stRNA-seq data and tumor-matched bulk RNA-seq data (n=15 matched samples). P value shown for Spearman rank coefficient. e. Mean spatially weighted correlation of spot-wise VI cluster enrichment scores across all stRNA-seq samples (n=15). [file media-4.pdf]

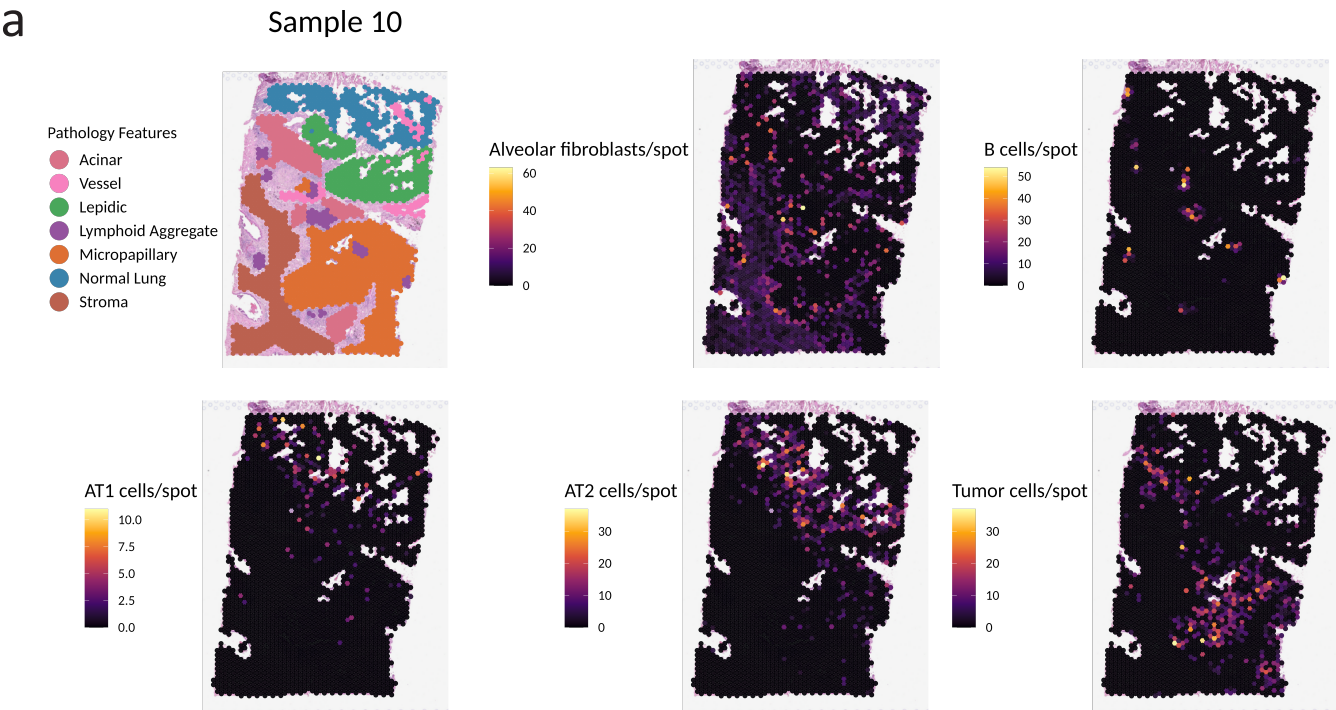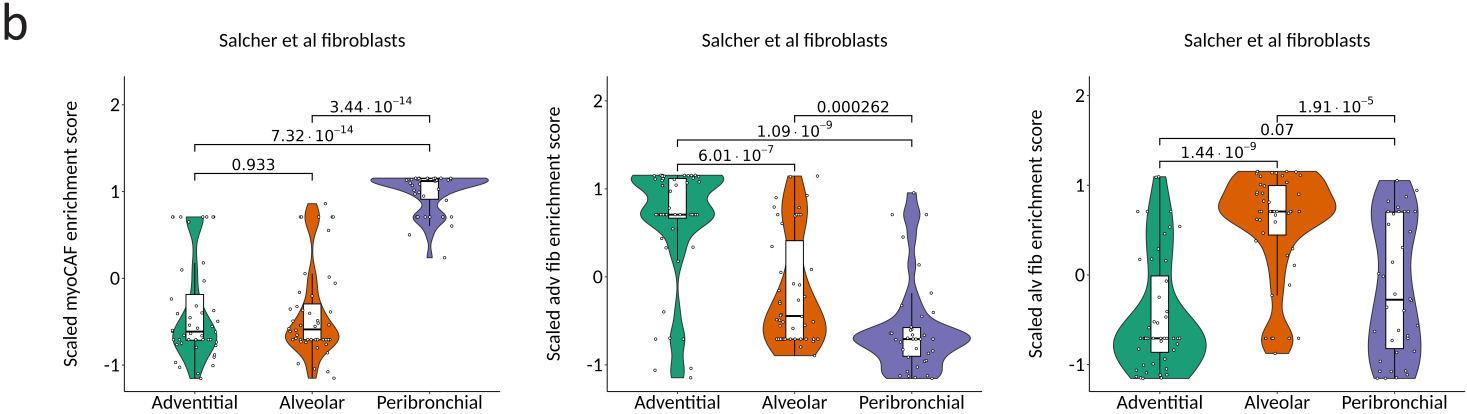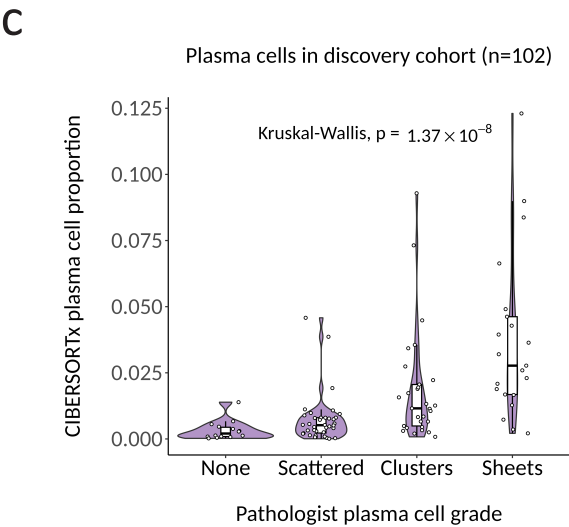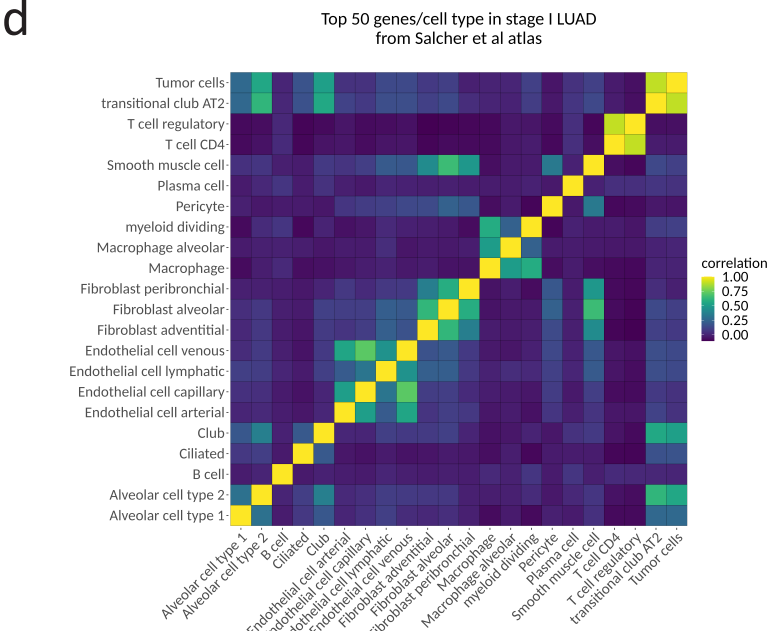

**Extended Data Fig. 5. Cell type deconvolution of stRNA-seq and bulk RNA-seq.**

Supplement: Supplement 5 — Extended Data Fig. 5. Cell type deconvolution of stRNA-seq and bulk RNA-seq. a. Representative images showing the association of cell type enrichment signatures with pathology. b. Sample-level enrichment scores (averaged over single cells) of NSCLC myofibroblastic-cancer associated, alveolar, and adventitial fibroblast signatures derived from Hanley et al. 2023 in fibroblast subpopulations from Salcher lung cancer atlas stage I LUAD samples. c. Proportions of plasma cells from deconvolution of the bulk RNA-seq discovery cohort, stratified by pathologist annotated plasma cell grade (n=102 with annotations). d. Correlation of cell type markers (using the average expression of the top 50 differentially expressed marker genes) in the stage I LUAD samples from the Salcher lung cancer atlas. [file media-5.pdf]

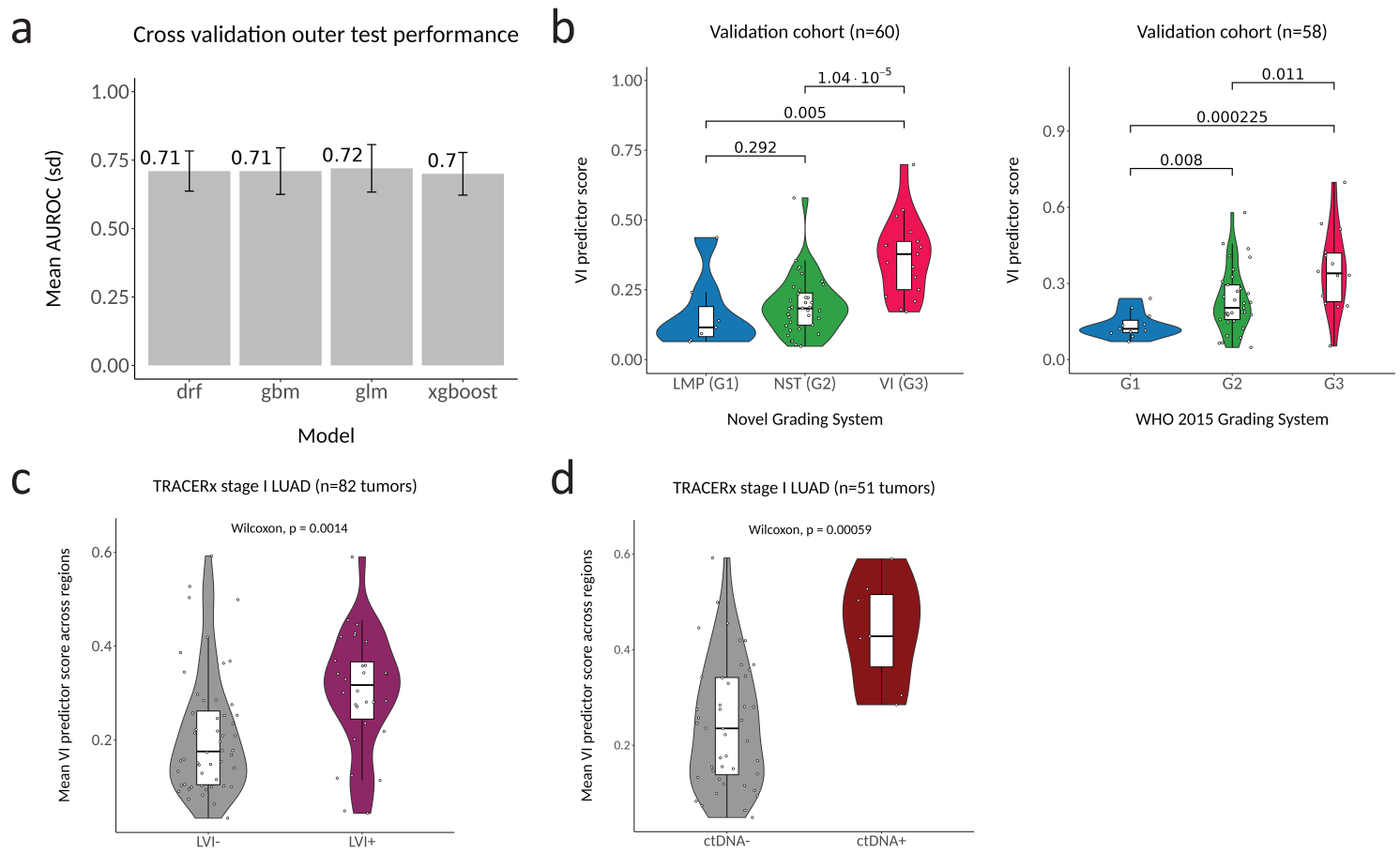

**Extended Data Figure 6. VI predictor development and validation.**

Supplement: Supplement 6 — Extended Data Fig. 6. VI predictor development and validation. a. Cross-validation performance for the four AutoML models in the outer test fold. Dr1, distributed random forest; gbm, gradient boosting machine; glm, generalized linear model; xgboost, extreme gradient boosting. Error bars represent standard deviation (SD) of AUROCs across all cross-validation folds (n=100). b. VI predictor association with novel and World Health Organization (WHO) 2015 grading systems in the validation cohort (n=60 tumors). For the WHO analysis, mucinous tumors (n=2) were removed from grade 3 as this grading system excludes mucinous tumors. P values were calculated by Wilcoxon test. c. Association of predictor scores with LVI+ tumors and d. patients’ preoperative ctDNA status in stage I LUAD RNA-seq data from the TRACERx cohort. [file media-6.pdf]
