# Supplementary material for: Identification of a gene expression signature of vascular invasion and recurrence in stage I lung adenocarcinoma via bulk and spatial transcriptomics
Source: bioRxiv. 2024 Jun 10:2024.06.07.597993. Preprint. [Version 1] doi: 10.1101/2024.06.07.597993 (PMC11195124; doi:10.1101/2024.06.07.597993)
Supplement: Supplement 2 — Extended Data Fig. 2. The four VI gene expression clusters individually predict VI in the discovery cohort even in the absence of LMP tumors. a. ROC curves for predicting VI+ vs. VI− tumors using the mean z-score of each gene expression cluster in the discovery cohort (n=103). b. ROC curves for predicting VI vs. NST tumors using the mean z-score of each gene expression cluster in the discovery cohort (n=80). LMP tumors were excluded from the analysis. P values are reported for the Wilcoxon test where the null hypothesis is that the AUROC is equal to 0.5. [file media-2.pdf]

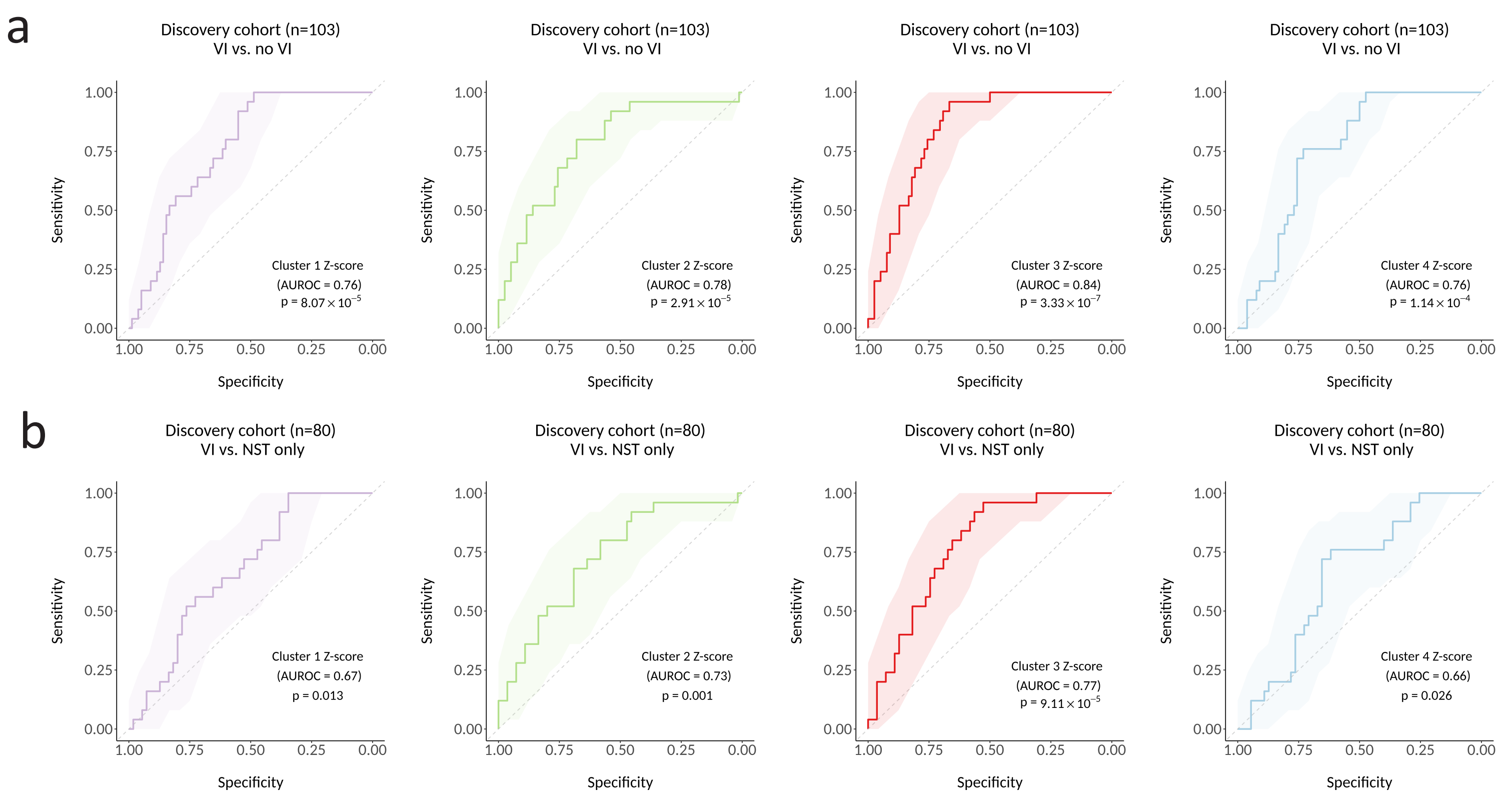

Extended Data Fig. 2. The four VI gene expression clusters individually predict VI in the discovery cohort even in the absence of LMP tumors.
