# Supplementary material for: Identification of a gene expression signature of vascular invasion and recurrence in stage I lung adenocarcinoma via bulk and spatial transcriptomics
Source: bioRxiv. 2024 Jun 10:2024.06.07.597993. Preprint. [Version 1] doi: 10.1101/2024.06.07.597993 (PMC11195124; doi:10.1101/2024.06.07.597993)
Supplement: Supplement 7 [file NIHPP2024.06.07.597993v1-supplement-7.pdf]

bioRxiv preprint doi: https://doi.org/10.1101/2024.06.07.597993; this version posted June 10, 2024. The copyright holder for this preprint (which was not certified by peer review) is the author/funder, who has granted bioRxiv a license to display the preprint in perpetuity. It is made available under aCC-BY 4.0 International license.

Supplementary Tables

Table S1. Clinical characteristics of resected stage I LUAD tumors with RNA-seq (post-QC) from the discovery cohort (n=103 tumors) using the novel histopathology classification.

	**LMP**	**NST**	**VI**	p value
**n**	23	55	25	
**Age (mean (SD))**	67.76 (11.39)	65.73 (10.11)	66.23 (7.92)	0.715
**Gender (%)**				0.099
Female	15 (65.2)	40 (72.7)	12 (48.0)	
Male	8 (34.8)	15 (27.3)	13 (52.0)	
**Race (%)**				0.177
Asian	2 (8.7)	2 (3.6)	1 (4.0)	
Black / African American	2 (8.7)	11 (20.0)	7 (28.0)	
Hispanic / Latino	2 (8.7)	0 (0.0)	0 (0.0)	
Unknown	0 (0.0)	1 (1.8)	1 (4.0)	
White	17 (73.9)	41 (74.5)	16 (64.0)	
**Pack years (mean (SD))**	35.50 (36.81)	43.77 (34.45)	36.37 (20.05)	0.494
**Smoking status (%)**				0.221
Current	4 (17.4)	18 (32.7)	12 (48.0)	
Former	13 (56.5)	30 (54.5)	11 (44.0)	
Never	6 (26.1)	6 (10.9)	2 (8.0)	
Unknown	0 (0.0)	1 (1.8)	0 (0.0)	
**Total size (cm) (mean (SD))**	1.34 (0.43)	1.75 (0.75)	2.00 (0.70)	0.005
**Invasive size (cm) (mean (SD))**	0.63 (0.46)	1.28 (0.60)	1.63 (0.57)	<0.001
**% Lepidic (mean (SD))**	63.48 (25.91)	25.27 (27.48)	16.60 (20.55)	<0.001
**% Acinar (mean (SD))**	23.48 (22.69)	26.45 (23.47)	36.00 (28.72)	0.169
**% Papillary (mean (SD))**	12.83 (24.90)	16.09 (23.23)	8.40 (9.76)	0.323
**% Solid (mean (SD))**	0.00 (0.00)	15.45 (30.60)	21.60 (32.04)	0.021
**% Micropapillary (mean (SD))**	0.00 (0.00)	6.00 (13.69)	8.60 (17.11)	0.068
**% Cribriform (mean (SD))**	0.22 (1.04)	10.55 (20.22)	8.80 (13.01)	0.038
**LI**	0 (0.0)	14 (25.5)	13 (52.0)	<0.001
**VPI**	0 (0.0)	11 (20.0)	13 (52.0)	<0.001

bioRxiv preprint doi: https://doi.org/10.1101/2024.06.07.597993; this version posted June 10, 2024. The copyright holder for this preprint (which was not certified by peer review) is the author/funder, who has granted bioRxiv a license to display the preprint in perpetuity. It is made available under aCC-BY 4.0 International license.

**STAS**	0 (0.0)	28 (50.9)	12 (48.0)	<0.001
**Institution (%)**				0.093
LHMC	6 (26.1)	5 (9.1)	6 (24.0)	
BMC	17 (73.9)	50 (90.9)	19 (76.0)	
**Procedure (%)**				0.23
Lobe	9 (39.1)	35 (63.6)	12 (48.0)	
Segment	2 (8.7)	1 (1.8)	2 (8.0)	
Wedge	12 (52.2)	19 (34.5)	11 (44.0)	

Note: The data are shown as the number and (%) unless otherwise indicated. Abbreviations: LMP, low malignant potential; NST, no special type; VI, vascular invasion; LI, lymphatic invasion; VPI, visceral pleural invasion; STAS, spread through air spaces; LHMC, Lahey Hospital and Medical Center; BMC, Boston Medical Center.

Table S2. Clinical characteristics of resected early-stage LUAD tumors with stRNA-seq (post-QC) from the discovery cohort (n=15 samples).

Sample ID	Batch	8th Stage	Novel Grade	Age	Sex	Smoking Status	RNA-seq	VI (Capture Frame)
1A	1	IB	VI	68	M	Former	Yes	1
1B	2	IB	VI	68	M	Former	Yes	0
2B	2	IA3	VI	68	F	Current	Yes	0
2A	2	IA3	VI	68	F	Current	Yes	0
3	2	IA1	NST	71	M	Former	Yes	0
4	2	IB	VI	66	M	Former	Yes	1
5	2	IA1	NST	72	M	Former	Yes	0
7	2	IA1	LMP	52	F	Current	Yes	0
8	1	IB	VI	66	M	Former	Yes	0
9	2	IA2	VI	63	F	Current	Yes	1
10	2	IA2	NST	69	M	Former	Yes	0
11	1	IIA	NST	63	F	Former	Yes	0
12	2	IB	NST	65	F	Former	Yes	0
13	1	IA1	NST	72	F	Former	Yes	0
14	2	IA2	NST	75	F	Former	Yes	0

Note: Abbreviations: LMP, low malignant potential; NST, no special type; VI, vascular invasion; LI, lymphatic invasion; VPI, visceral pleural invasion; STAS, spread through air spaces; LHMC, Lahey Hospital and Medical Center; BMC, Boston Medical Center.

Table S3. Clinical characteristics of resected stage I LUAD tumors with RNA-seq (post-QC) from the validation cohort (n=60 tumors) using the novel histopathology classification.

	**LMP**	**NST**	**VI**	p value
**n**	7	36	17	
**Age (mean (SD))**	65.57 (8.50)	66.31 (7.30)	61.76 (6.44)	0.107

bioRxiv preprint doi: https://doi.org/10.1101/2024.06.07.597993; this version posted June 10, 2024. The copyright holder for this preprint (which was not certified by peer review) is the author/funder, who has granted bioRxiv a license to display the preprint in perpetuity. It is made available under aCC-BY 4.0 International license.

**Gender (%)**				0.205
Female	6 (85.7)	19 (52.8)	8 (47.1)	
Male	1 (14.3)	17 (47.2)	9 (52.9)	
**Pack years (mean (SD))**	31.43 (11.80)	49.53 (25.19)	51.65 (17.91)	0.114
**Smoking status (%)**				0.408
Current	2 (28.6)	13 (36.1)	9 (52.9)	
Former	5 (71.4)	23 (63.9)	8 (47.1)	
**Total size (cm) (mean (SD))**	1.90 (0.76)	1.93 (0.87)	2.22 (0.90)	0.484
**Invasive size (cm) (mean (SD))**	0.81 (0.38)	1.68 (0.95)	2.06 (0.84)	0.01
**% Lepidic (mean (SD))**	55.71 (22.25)	22.78 (29.46)	9.12 (14.71)	0.001
**% Acinar (mean (SD))**	26.43 (22.12)	29.31 (26.68)	20.29 (24.46)	0.494
**% Papillary (mean (SD))**	17.86 (30.80)	19.44 (28.63)	8.82 (10.97)	0.361
**% Solid (mean (SD))**	0.00 (0.00)	5.00 (11.46)	46.47 (37.03)	<0.001
**% Micropapillary (mean (SD))**	0.00 (0.00)	10.28 (20.91)	4.71 (10.23)	0.267
**% Cribriform (mean (SD))**	0.00 (0.00)	13.33 (27.67)	10.59 (20.30)	0.415
**LI (%)**	0 (0.0)	17 (47.2)	9 (52.9)	0.045
**VPI (%)**	0 (0.0)	5 (13.9)	7 (41.2)	0.025
**STAS (%)**	0 (0.0)	14 (38.9)	10 (58.8)	0.027
**Procedure (%)**				0.79
Lobe	6 (85.7)	27 (79.4)	14 (82.4)	
Segment	1 (14.3)	2 (5.9)	1 (5.9)	
Wedge	0 (0.0)	5 (14.7)	2 (11.8)	

Note: Abbreviations: LMP, low malignant potential; NST, no special type; VI, vascular invasion; LI, lymphatic invasion; VPI, visceral pleural invasion; STAS, spread through air spaces; all cases were from Lahey Hospital and Medical Center.
